# Twelve Lessons on the Structures of the Central Nervous System for Physicians and Surgeons

**Published:** 1891-05

**Authors:** 


Twelve Lessons on the Structure of the Central Nervous System for Physicians and Students. By Dr. Ludwig Edinger, Frankfort-on-the-Main. Second revised edition, with 133 illustrations. Translated by Willis Hall Vittum, M. D., St. Paul, Minn. Edited by C. Eugene Riggs, A. M., M. D., Professor of Mental and Nervous Diseases, University of Minnesota; member of the American Neurological Association. 8vo, pp. xii.—230. Philadelphia and London: F. A. Davis. 1890. Price, $1.75 net.

This is an excellent translation of Prof. Edinger's well-known work on the Anatomy of the Nervous System. The editor's prominence in American neurological circles tends to make the book more authoritative and reliable, especially on this side of the Atlantic. Many of the new terms employed in anatomical nomenclature have been used by the editor and translator, and it is to be hoped that in the preparation of the Second American Edition still more may find their way into its pages. The 133 illustrations are taken from the German edition.

The typography is up to the high standard of the publisher, and adds much to the beauty of the book.

